# Low-Temperature Hydrophilic Pervaporation of Lactic Acid Esterification Reaction Media

**DOI:** 10.3390/membranes12010096

**Published:** 2022-01-17

**Authors:** Elena González Díaz, Sonia Álvarez-García, Susana Luque, José R. Álvarez

**Affiliations:** 1 Sulzer Chemtech Ltd., 4123 Allschwil, Switzerland; elena.gonzalez@sulzer.com; 2 Department of Chemical and Environmental Engineering, University of Oviedo, 33071 Oviedo, Spain; alvarezsonia@uniovi.es (S.Á.-G.); sluque@uniovi.es (S.L.)

**Keywords:** pervaporation, hydrophilic membrane, temperature

## Abstract

Esterification reactions show a limited conversion due to the presence of water, which favors the opposite reaction. The removal of water from the reaction mixture increases the production of the ester. Pervaporation is an effective dehydration technique, usually applied to binary mixtures. The effect on pervaporation of a reactive multicomponent system involving water, ethanol, ethyl lactate and lactic acid with high acid concentration (13.5 wt. %) at relatively low temperatures (40–80 °C) was studied. Three hydrophilic membranes mainly fabricated for dehydration purposes from Sulzer Chemtech were used, i.e., PERVAP™ 3100, PERVAP™ 2216 and PERVAP™ 1131. The last one revealed as the most suitable for the application and it was further characterized with binary and ternary solutions. The membrane showed high affinity for the lactic acid. The acid permeation played a key role in the water/ethanol and water/ethyl lactate selectivity. Lactic acid permeates and crystalizes in the permeate side of the membrane at very low water concentration (below 2 wt. %), causing a drop in flux and membrane selectivity. Ethyl lactate is responsible of the loss of integrity of the membranes.

## 1. Introduction

The production of lactic acid esters is one the most promising uses of lactic acid, since they can be used in the pharmaceutical, food, automotive and aerospace industries. Lactic esters have a great potential as green solvents, because of their high boiling points and nontoxic and biodegradable properties, replacing common organic solvents like methyl isobutyl ketone [1].

Lactic acid esterification is commonly catalyzed by organic and inorganic acids, ionic exchange resins and zeolites [2], but enzymes as lipases have also been employed [3]. Water content is a key parameter that affects the equilibrium concentration of reactants and products in the reaction medium, because it favors the ester hydrolysis and limits the yield of the ester. Moreover, in the case of enzyme-catalyzed esterification, it also affects the thermal inactivation of the enzymes. 

Pervaporation (PV) is an attractive method for water removal in esterification reactions, helping the system to shift the conversion beyond the equilibrium limitation [4,5]. Hydrophilic membranes, i.e., those able to separate mainly water from an organic–water mixture, are employed. Membrane common materials are: polyvinylalcohol (PVA), polyvinilalcohol/polyacrilonitrile (PVA/PAN), polyetherimide (PEI) [6], with PVA the most frequently employed [7]. The use of nonpolymeric materials such as zeolites is also remarkable [8,9,10].

One of the first applications in esterification reactions was introduced in 1987 by Kita et al. [11] who found that PV was able to shift the conversion of oleic acid with ethanol beyond the reaction equilibrium value and, therefore, reduce the need for excess reactants. The first kinetic studies on the coupling of PV and esterification were published by David et al. [12,13] in the reaction of propionic acid with 1-propanol and 2-propanol using a PVA membrane. The results showed yield improvements of 20%.

Bagnell et al. [14] used catalytically active PV Nafion tubes in the reaction of acetic acid with methanol and *n*-butanol, increasing the conversions from 73% and 70% to 77% and 95%, respectively. Butyl acetate was also obtained by Zhu and Chen [15] with a catalytic crosslinked PVA active layer on a porous ceramic plate support; by Xuehei and Lefu [16] with a phosphatic PVA/PAN membrane; by Liu and Chen [17] with a PVA/porous ceramic one; by Peters et al. [18] with a catalytic zeolite coat on a ceramic hollow fiber silica support; by Qing et al. [19] with a layer of PVA and zirconium sulfate tetrahydrate on a PVA/polyethersulfone membrane; and by Zhang et al. [20] with a coat of PVA and ion-exchange resins on a dense PVA/polyethersulfone membrane.

Benedict et al. [5,21] esterified succinic and lactic acid with ethanol in the presence of solid catalysts (Amberlyst Xn-1010 and Nafion NR50) using GFT-1005 and T1-b pervaporation membranes. The esterification of lactic acid and ethanol in the presence of Amberlyst 15 was done by Ma et al. [22] with a chitosan–tetraethoxysilane organic–inorganic hybrid membrane, by Delgado et al. [23] with the hydrophilic PERVAP™ 2201, by Pereira et al. [24] with a microporous silica membrane, and by Zhang et al. [25] with three home-made composite membranes of glutaral, chitosan, gelatin, carbomer, hyaluronic acid and carbomer on PAN. Budd et al. [26] chose zeolite/polyelectrolyte multilayer membranes and *p*-toluene sulfonic acid for obtaining ethyl lactate. This acid and the PERVAP™ 2205 membrane were combined with an ionic solvent to produce bornyl acetate from acetic acid and borneol by Izák et al. [27]. Amberlyst-15 and PERVAP™ 2201 were also employed by Assabumrungrat et al. [28] in the synthesis of methyl acetate and by Sanz and Gmehling [29,30] in the reaction of acetic acid with isopropanol.

The aim of this work is to find an adequate PV membrane (initially designed for the dehydration of solvents) for the dehydration of the mixture involved in the esterification of lactic acid with excess ethanol. The chosen initial acid to alcohol molar ratio was between 1:5 and 1:10, the pH is close to 3 units and the initial amount of water is around 10 wt. %, since aqueous solutions of lactic acid (80 wt. %) and azeotropic ethanol were used, aiming to conduct a study with mixtures close to the actual industrial reaction conditions. Concentration ranges differ considerably from the study of Delgado et al. [31], in which the water content was always higher than 10 wt. %. In the present study, water concentration was reduced to 2 wt. % and below.

After a preliminary screening at 60 °C using three hydrophilic membranes, a more thorough study was performed on the most suitable membrane using different feed composition (binary, ternary, and quaternary solutions) and two other temperatures (40 °C, the optimum temperature for biocatalysis, and 80 °C, closer to the usual working temperature for dehydration by PV).

## 2. Materials and Methods

### 2.1. Materials and Analytical Methods

L(+) Lactic acid from Riedel de Häen (80 wt. % aqueous solution; Seelze, Germany), ethyl lactate (99%, Fluka, Buchs, Switzerland), and azeotropic ethanol (96%, Panreac, Barcelona, Spain) were all of analytical grade and used without further purification. 

Ethyl lactate, ethanol, and water concentration in permeate were measured by gas chromatography with a thermal conductivity detector (GC-TCD Shimatzu GC-8, Kyoto, Japan), using a Supelco 30 m capillary column. Injection temperature was 200 °C and the column was kept at 190 °C. 

Water concentration in feed was analyzed by a Karl Fischer coulometer (Metrohm 737 KF, Herisau, Switzerland). Lactic acid concentration in feed and permeate was analyzed by titration with NaOH 0.05 M, using phenolphthalein as indicator.

All concentrations are expressed as weight per cent.

### 2.2. Pervaporation Membranes

In this work, the commercial PERVAP™ 2216 and the experimental PERVAP™ 1131 and PERVAP™ 3100 membranes, manufactured by Sulzer Chemtech Ltd. (Neunkirchen, Germany) [32], were tested. These membranes were chosen after a preliminary screening of several commercial PV membranes (results not shown), most of which had severe damage within a short time when exposed to concentrated lactic acid/ethyl lactate aqueous solutions. 

PERVAP™ membranes are asymmetric composite membranes for water permeation. PERVAP™ 2216 membrane is made of crosslinked PVA supported on an ultrafiltration membrane made of PAN, which is cast on a layer of non-woven porous polyester. PVA has relatively good chemical stability, film-forming ability, and high hydrophilicity, but has a poor stability in aqueous solutions. Therefore, it needs to be insolubilized by crosslinking (with maleic acid, glutaraldehyde, etc.) or otherwise be modified to get good mechanical properties and selective permeability towards water. This membrane is similar to other commercial PV membranes, such as PERVAP™ 2201D, PERVAP™ 2200, PERVAP™ 2201 or PERVAP™ 2211, differences being in the degree of crosslinking [33]. 

PERVAP™ 3100 is similar to PERVAP™ 2216 made with a thin top layer and an additional nonwoven supporting structure of polypropylene, which aims to provide higher fluxes while maintaining good mechanical resistance.

PERVAP™ 1131 is an experimental membrane, based on polyethylene polymerization by plasma application, which makes it more resistant towards acid aqueous solutions. 

According to the manufacturer, the three membranes are designed to work between 40 and 105 °C.

### 2.3. Pervaporation Apparatus and Procedure

PV experiments were carried out in a commercially available test equipment from Sulzer Chemtech, a description of which can be found elsewhere [34]. The effective membrane area of the test cell was 178 cm^2^ and the volume of the feed tank was approximately 2 L. In the feed side, an additional water cooler was also used when the required temperature was 40 °C. The permeate side pressure was always maintained at 11 ± 1 mbar. Two cold traps of dry ice in ethanol located in the permeate line allowed the condensation and collection of permeate samples. Prior to be used in the PV experiments, the membranes were placed in the test cell and contacted with the feed solution for at least 16 h. During this conditioning, permeate pressure was always kept at 11 mbar.

Feed was pumped to the membrane cell at a flow rate of 40 L/h ensuring a turbulent flow (Reynolds number of 3000), to achieve a similar hydrodynamic regime as in industrial modules. 

Four feed mixtures were employed to assess the influence of each component and the possible coupling effects. The initial concentrations of water, lactic acid and ethyl lactate are always the same in the feed stream, while the ethanol is adjusted accordingly:

- Binary mixture: 10 wt. % water and 90 wt. % ethanol.
- Ternary mixtures: One with ester, water, and alcohol (17.7 wt. % ethyl lactate, 10 wt. % water and 72.3 wt. % ethanol) and another with acid, water, and alcohol (13.5 wt. % lactic acid, 10 wt. % water and 76.5 wt. % ethanol). There is no lactic acid + ethyl lactate + ethanol mixture because ethanol and lactic acid were used from commercial aqueous solutions, as described in Section 2.1.
- Quaternary mixture: 13.5 wt. % lactic acid, 17.7 wt. % ethyl lactate, 10 wt. % water and 58.8 wt. % ethanol.

Experiments proceeded until values below 2 wt. % of water in the feed were achieved, which corresponded to almost no permeation through the membrane. Concentration profiles for all the compounds were followed by sampling feed and permeate and analyzing them as described previously. Permeation experiments were on average week-long tests (except for the 22,016 membrane that spanned twice as long). Some tests were replicated and the errors were below 2%.

The separation factor (*β*) was calculated according to the ratio of concentrations between permeate (*p*) and feed (*f*) for two substances (*i* and *j*) with the following expression:


(1) $$\beta_{pervap\;i,j}=\frac{C_{pi}}{C_{pj}}\frac{C_{fj}}{C_{fi}}$$

Wijmans and Baker [35] considered pervaporation as a combination of evaporation and membrane separation steps, and therefore including two contributions in the separation factor: *β_pervap_ = β_evap_·β_mem_*, where *β_evap_* is the separation factor resulting of the vapor-liquid equilibrium:

(2) $$\beta_{evap\;i,j}=\frac{p_{i}^{'}}{C_{fi}}\frac{C_{fj}}{p_{j}^{'}}$$

and *β_mem_* is the separation factor due to the membrane. *β_mem_* can be expressed, in turn, as the ratio of the permeate fluxes (*J*) and partial pressures (*p*’) of the compounds *i* and *j* in the feed. 

The flux of an *i* component is described as a function of its permeance (P*_i_*) times the partial pressure difference between the feed (*p*′_*i*_) and the permeate (*p*″_*i*_):


(3) $$J_{i}=P_{i}(p_{i}^{'}-p_{i}^{"})$$

Partial pressures in the feed side are calculated using liquid composition, pure component vapor pressure (calculated using an extended Antoine equation, found in the Appendix A) and the activity coefficient, which was obtained from the UNIQUAC activity model (the parameters are also listed in the Appendix A) which proved to be the best model for this system, based on the multicomponent vapor-liquid equilibria data experimentally determined by Delgado et al. [36]. Activity coefficients are close to one for both lactic acid and ethanol, between 1.7 and 2 for water, and between 6 and 7 for ethyl lactate, depending on the composition. The partial pressures in the permeate can be calculated directly from the total permeate pressure times the permeate concentration. 

The ratio of permeate fluxes between components *i* and *j* is


(4) $$\frac{J_{i}}{J_{j}}=\frac{P_{i}}{P_{j}}\;\frac{\left(p_{i}^{'}-p_{i}^{"}\right)}{\left(p_{j}^{'}-p_{j}^{"}\right)}$$

As the ratio of permeances (P*_i_*/P*_j_*) is the *i*/*j* membrane selectivity (*α_ij_*), and the ratio of fluxes is equal to the ratio of permeate pressures, Equation (4) leads to


(5) $$\frac{J_{i}}{J_{j}}=\frac{p_{i}^{"}}{p_{j}^{"}}=\alpha_{ij}\;\frac{\left(p_{i}^{'}-p_{i}^{"}\right)}{\left(p_{j}^{'}-p_{j}^{"}\right)}$$

Finally, combining (1), (2) and (5), the resulting expression is


(6) $$\beta_{pervap}=\;\beta_{evap}\;\alpha_{ij}\frac{\left(p_{i}^{'}-p_{i}^{"}\right)}{\left(p_{j}^{'}-p_{j}^{"}\right)}\;\frac{p_{j}^{'}}{p_{i}^{'}}$$

If permeate pressure is considerably lower than the feed pressure, it simplifies to:


(7) $$\beta_{pervap}=\;\beta_{evap}\;\alpha_{ij}$$

Equation (7) will be used in this study. $\beta_{pervap}$ is obtained experimentally, $\beta_{evap}$ is calculated from vapour-liquid equilibria, and $\alpha_{ij}$ will be ratio between them.

## 3. Results

### 3.1. Membrane Selection

To select the most suitable membrane for the mixture representing the reaction to produce ethyl lactate, a 13.5 wt. % lactic acid and 17.7 wt. % ethyl lactate solution in ethanol (acid to alcohol molar ratio of 1:10), with 10 wt. % of water content, was pumped to the membrane cell. 

**Figure 1:**
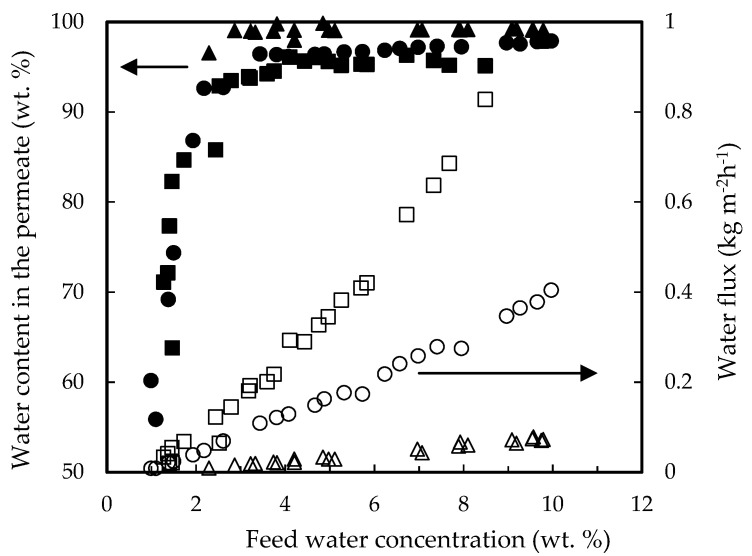
Water permeation fluxes (open symbols) and water concentration in permeate (filled symbols) as a function of feed water content for the three PV membranes tested. (■,☐) PERVAP™ 3100, (●,○) PERVAP™ 1131 and (▲,△) PERVAP™ 2216. Initial feed content: 13.5% lactic acid, 17.7% ethyl lactate, 10% water and 58.8% ethanol. Temperature = 60 °C.

Figure 1 shows the influence of the feed water concentration on the permeate water flux for the three membranes tested. PERVAP™ 2216 gave the lowest flux values, in fact more than 12 days were necessary to reduce the feed water content from 10 to 2 wt. %. However, this membrane shows the best selectivity, with water contents in the permeate above 99.5 wt. %. This is coherent with a highly cross-linked top layer (and likely thicker than the other two). The selectivity of the other two membranes was very similar, although the flux of the PERVAP™ 3100 was twice as much that of PERVAP™ 1131. However, the former had a very poor chemical resistance and had to be replaced weekly during the study. Its thinner top layer was not robust enough for this application.

**Figure 2:**
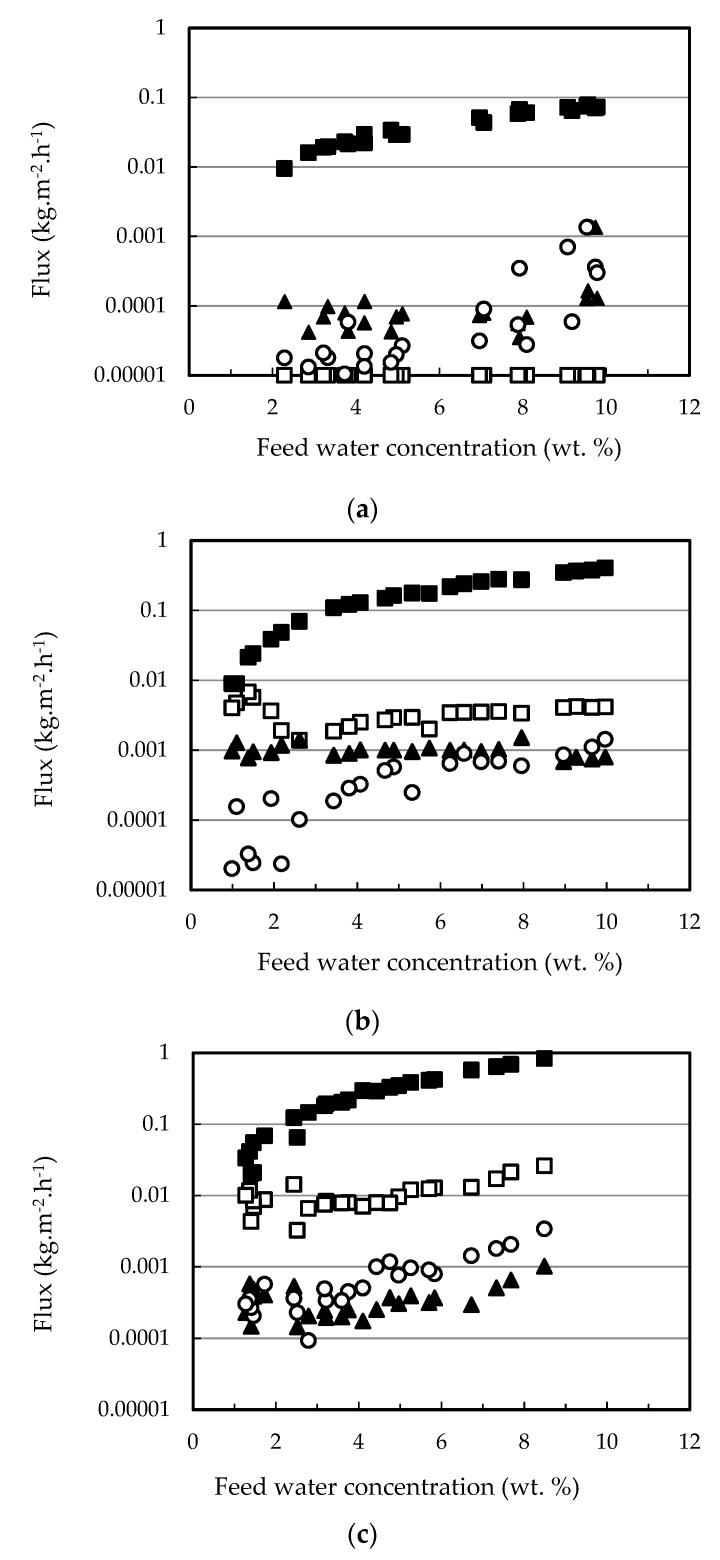
Individual component fluxes as a function of feed water content for the three PV membranes tested: (**a**) PERVAP™ 2116, (**b**) PERVAP™ 1131, (**c**) PERVAP™ 3100. (■) Water, (☐) ethanol, (▲) ethyl lactate, (○) lactic acid. Initial feed content: 13.5% lactic acid, 17.7% ethyl lactate, 10% water and 58.8% ethanol. Temperature = 60 °C.

**Figure 3:**
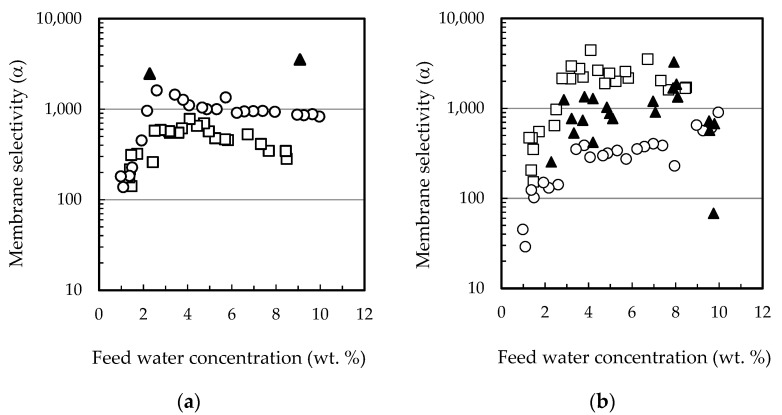
Membrane selectivity as a function of feed water content for the three PV membranes tested (**a**) water/ethanol, (**b**) water/ethyl lactate. (☐) PERVAP™ 3100, (○) PERVAP™ 1131 (▲) PERVAP™ 2216. Initial feed content: 13.5% lactic acid, 17.7% ethyl lactate, 10% water and 58.8% ethanol. Temperature = 60 °C.

The permeation fluxes of all the components and the selectivity profiles water/ethanol and water/ethyl lactate as a function of the water content in the feed mixture are shown in Figure 2 and Figure 3, respectively. Ethanol fluxes corresponding to the PERVAP™ 2216 were below the detection limit (under 0.005 wt. %), and thus, only two non-infinite selectivity values could be calculated. Component fluxes, as shown in Figure 2, follow the trend: water > ethanol > ethyl lactate ≈ lactic acid, in accordance with the molecular size. The molecular weights of water, ethanol, lactic acid, and ethyl lactate are 18.01, 46.07, 90.08, and 118.13 g/mol, respectively.

With respect to the membranes, the permeation flux decreased in the order PERVAP™ 3100 > PERVAP™ 1131 > PERVAP™ 2216. Moreover, two differentiated behaviors could be observed depending on the water content in the feed:

- Above 2 wt. %: The fluxes for all feed components increased linearly as the water concentration in the feed increased (Figure 2). There was an order of magnitude difference between water and ethanol fluxes, and almost another order of magnitude between ethanol and both ethyl lactate and lactic acid fluxes, respectively. Selectivity of water/ethanol and ethyl lactate/ethanol (Figure 3) was constant.

- Below 2 wt. %: Water concentration in the permeate dropped exponentially when water content in the feed decreased below 2 wt. %. PERVAP™ 2216 fluxes could be considered negligible, even before reaching 2 wt. % (Figure 1 and Figure 2a). PERVAP™ 1131 showed a constant ethanol and ethyl lactate fluxes, while lactic acid flux decreased dramatically like water flux (Figure 2b). There was, however, significant scattering below 2 wt. %. Finally, PERVAP™ 3100 also showed a sharp decrease in the selectivity of water/ethanol and water/ethyl lactate as the water feed content was low.

This behavior has been previously reported in literature [37] and can be explained considering the effect of different substances in membrane swelling.

### 3.2. PERVAP™ 1131 Membrane Characterization

Due to the low water flux of PERVAP™ 2216, to the poor resistance of PERVAP™ 3100, PERVAP™ 1131, which was acid-resistant and gave high selectivity, was selected as the most appropriate membrane for further analysis. The performance of PERVAP™ 1131 with different feed composition and temperatures was, thus, studied more thoroughly.

In the case of feed composition, four systems were employed to assess the influence of each component and the possible coupling effects: one binary, two ternary and the quaternary reaction mixture used for membrane screening. 

**Figure 4:**
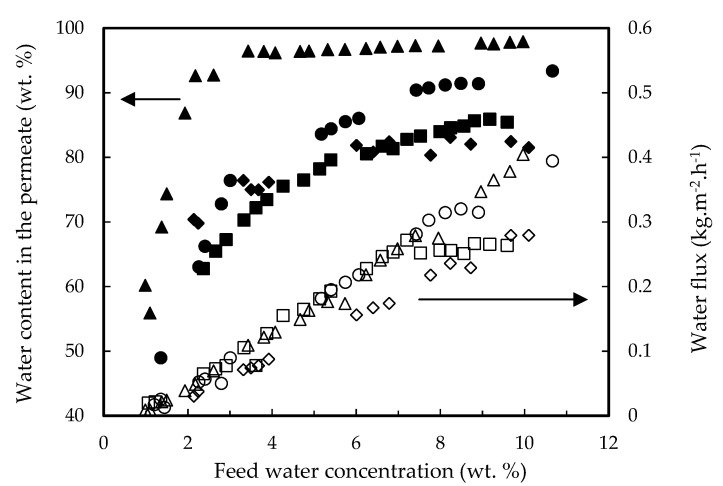
Water permeation fluxes (open symbols) and water concentration in permeate (closed symbols) as a function of feed water content for four mixtures: (▲,△) 13.5% lactic acid, 17.7% ethyl lactate, 10% water and 58.8% ethanol; (■,☐) 17.7% ethyl lactate, 10% water and 72.3% ethanol; (◆,◇) 13.5% lactic acid, 10% water and 76.5% ethanol; (●,○) 10% water and 90% ethanol. PERVAP™ 1131 membrane at T = 60 °C.

Figure 4 shows fluxes and water permeate contents for the four above-mentioned mixtures. The highest content of water in permeate corresponded to the quaternary mixture, but sharply dropped when feed water decreases below 2 wt. %. This was also found but less pronounced in the other mixtures. The flux of water was the same independently of the feed mixture tested. 

**Figure 5:**
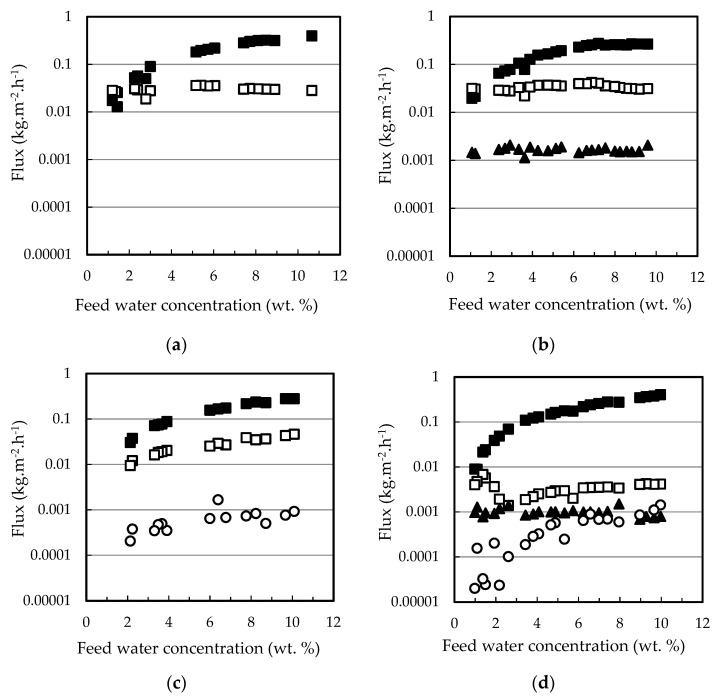
Individual component fluxes as a function of feed water content for mixtures of different composition: (**a**) Water + ethanol, (**b**) water + ethanol + ethyl lactate, (**c**) water + ethanol + lactic acid, (**d**) water + ethanol + ethyl lactate + lactic acid. (■) Water, (☐) ethanol, (▲) ethyl lactate, (○) lactic acid. PERVAP™ 1131 membrane at T = 60 °C.

**Figure 6:**
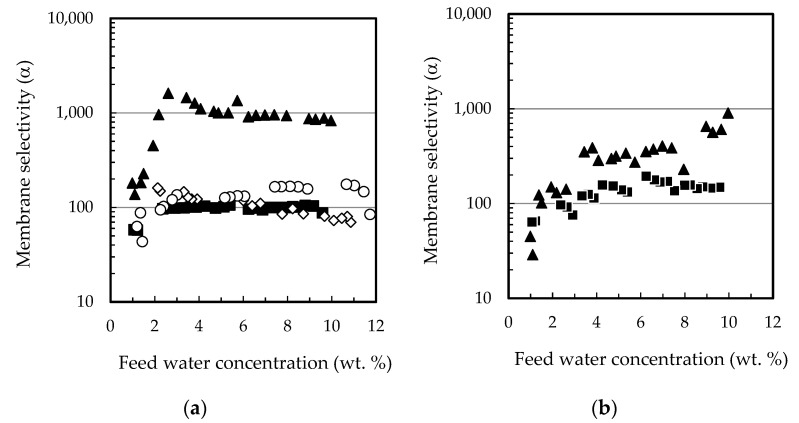
Selectivity as a function of feed water content for mixtures of different composition: (**a**) water/ethanol, (**b**) water/ethyl lactate. (▲) contains 13.5% lactic acid, 17.7% ethyl lactate, 10% water and 58.8% ethanol; (■) 17.7% ethyl lactate, 10% water and 72.3% ethanol; (◇) 13.5% lactic acid, 10% water and 76.5% ethanol; (○) 10% water and 90% ethanol. PERVAP™ 1131 membrane at T = 60 °C.

Figure 5 shows the influence of the different substances when added to a water-ethanol mixture. Figure 5a shows that the flux of water was initially one order of magnitude higher than the flux of ethanol, that stays relatively constant. However, both fluxes became very similar as the feed water content dropped down to 2 wt. %. When ethyl lactate was added as third component there was no effect on water or ethanol fluxes with respect to the binary mixture, and the flux of ethyl acetate was two orders of magnitude lower than water, as shown in Figure 5b. In contrast, when the third component was lactic acid, the flux of ethanol decreased at the same rate as that of water, the flux of lactic acid being two to three orders of magnitude lower, as shown in Figure 5c. When the four compounds are in the mixture (Figure 5d), there was a combined effect of lactic acid and ethyl lactate in the flux of ethanol. The latter was at that point two orders of magnitude lower than the flux of water until the water feed content dropped to 2 wt. %, at which point it sharply increased to equal that of water. Lactic acid flux also decreased more significantly when the water feed content dropped down 5 wt. %. On the other hand, the flux of ethyl lactate stayed constant, as in the tertiary mixture shown in Figure 5b. The resulting water/ethanol and water/ethyl lactate membrane selectivity is shown in Figure 6 as a function of water feed composition.

Figure 6a shows that the water/ethanol selectivity for binary and ternary mixtures was the same for all water feed compositions, and the selectivity for the quaternary mixture was one order of magnitude higher in favor of water permeation, which indicates a strong effect of the presence at the same time of both lactic acid and ethyl lactate, reducing ethanol permeation by an order of magnitude as mentioned. When ethyl lactate was added to water-ethanol mixture, there was no change of membrane selectivity, as described from Figure 5a,b, and water and ethanol permeation rates were about the same. When lactic acid was added to water-ethanol mixture, there was an increase in water-ethanol selectivity at low feed water concentration, because both water and ethanol fluxes decreased unlike in the other cases, as shown in Figure 5c. When lactic acid and ethyl lactate were both added to the water-ethanol mixture, the water/ethanol selectivity increased tenfold because lactic acid and ethyl lactate were preferentially dissolved in the membrane, allowing the passage of water but hindering the passage of ethanol (this will be further analyzed in Section 3.3). Moreover, water-ethyl lactate selectivity was not affected (Figure 6b), as expected from the fluxes shown in Figure 5b,d. 

Membranes were not fouled by any of the components of the mixture. As shown in Figure 4 water permeation fluxes were the same for any of the binary, ternary or quaternary mixtures. The same membrane was used for all experiments and no cleaning was performed between the tests. 

Nevertheless, a relative long exposure of the membrane (30 days) to the ethyl lactate showed membrane deterioration (coloring and the presence of pores or defects in the surface), in fact the membrane started to dissolve, due to the combined action of acid and the ethyl lactate as solvent, losing its integrity.

### 3.3. Degree of Swelling

To study the interaction of all the components with the membrane, the degree of membrane swelling when subjected to static contact was determined. Swelling depends not only on the structure of membrane but also on the affinity of the components towards the membrane, the interactions between components, and the way the interactions of each component with the membrane affects the interaction of the rest of components, as expected from a nonideal mixture [38,39,40]. 

The degree of swelling was calculated for all compounds according to the variation in the weight of a piece of membrane placed in a vial for 15 days at 60 °C, as follows:

(8) $$Degree\;of\;Swelling\;(\%)=\frac{W_{w}-W_{d}}{W_{d}}\;100$$

where *W_w_* and *W_d_* are the weights of the membrane in wet and dry states, respectively.

Pure lactic acid and ethyl lactate are the components with more affinity for the membrane, the degree of swelling for lactic acid being twice the value for ethyl lactate (Table 1). For the other components, only water showed some relevant swelling, but five times lower than ethyl lactate. This suggests a very strong affinity of the PERVAP™ 1131 towards acids. To verify this hypothesis, several pieces of membrane were soaked with water, ethyl lactate and water respectively at 60 °C, adding 0.5 mL of concentrated sulfuric acid (to have a pH below 2 units, as with the lactic acid). The results show an increase in swelling in accordance with the suggested acid affinity.

**Figure 7:**
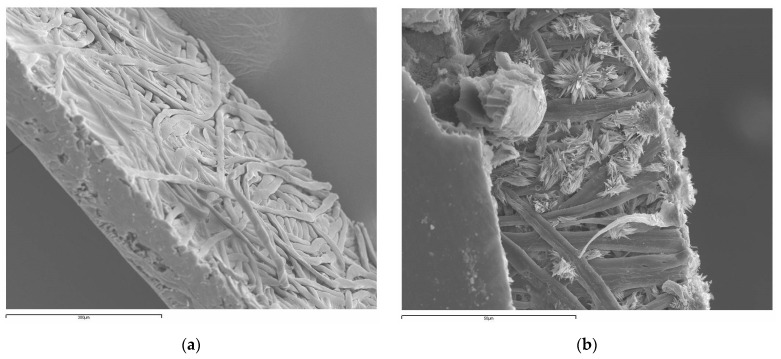
PERVAP™ 1131 membrane SEM pictures: (**a**) new membrane, (**b**) membrane with lactic acid crystals after pervaporation.

Comparing the degree of swelling of the four solutions in the characterization of the PERVAP™ 1131, the highest value was obtained for the four-component solution, which is the actual case in an esterification reaction and the one which yields the highest selectivity for water. Figure 6a shows, as mentioned, the higher water/ethanol selectivity, which clearly accounts for the effect of lactic acid in competition with the ethyl lactate. In fact, it is the combined effect of the acid and the ethyl lactate responsible for that change. Moreover, during the pervaporation experiments, due to the high permeate rate of water with respect to the rest of the components, there is a surprising reduction of lactic acid concentration in the feed, while its concentration should increase, being the least permeable. The lactic acid disappearance cannot be justified by the esterification reaction since there is no match in the mass balance that accounts for ethanol or ethyl lactate variations. This, together with the high degree of interaction, indicates that the acid that disappears from the solution permeates the membrane. This was found to happen at the permeate side of the membrane, where the vacuum conditions result in reduced water and ethanol concentrations and gave rise to the formation of lactic acid crystals, as proven by microscopy (Figure 7). The reduction of water and the presence of ethyl lactate in the membrane promotes the crystallization of lactic acid in a nonaqueous environment.

### 3.4. Temperature Effect

**Figure 8:**
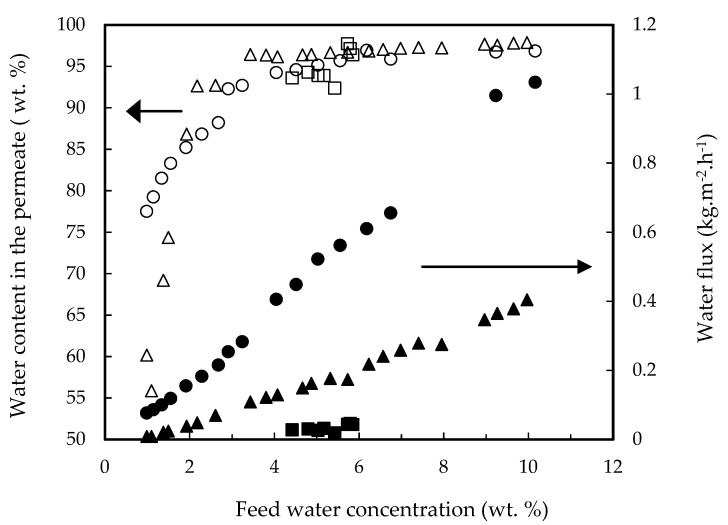
Water permeation fluxes (open symbols) and water concentration in permeate (closed symbols) as a function of feed water content for the PERVAP™ 1131 membrane at different temperatures: (●,○) T = 80 °C, (▲,△) T = 60 °C, (■,☐) T = 40 °C. Initial feed content: 13.5% lactic acid, 17.7% ethyl lactate, 10% water and 58.8% ethanol.

Experiments were also performed at 40 and 80 °C. Figure 8 shows the water permeation fluxes together with the water content in the permeate. Data at 40 °C were scarce due to the low permeation rate. Temperature clearly increases water flux, and the water permeate content sharply decreases when the water feed content drops down 2 wt. %, as it was shown for the three membranes tested in Figure 1. 

**Figure 9:**
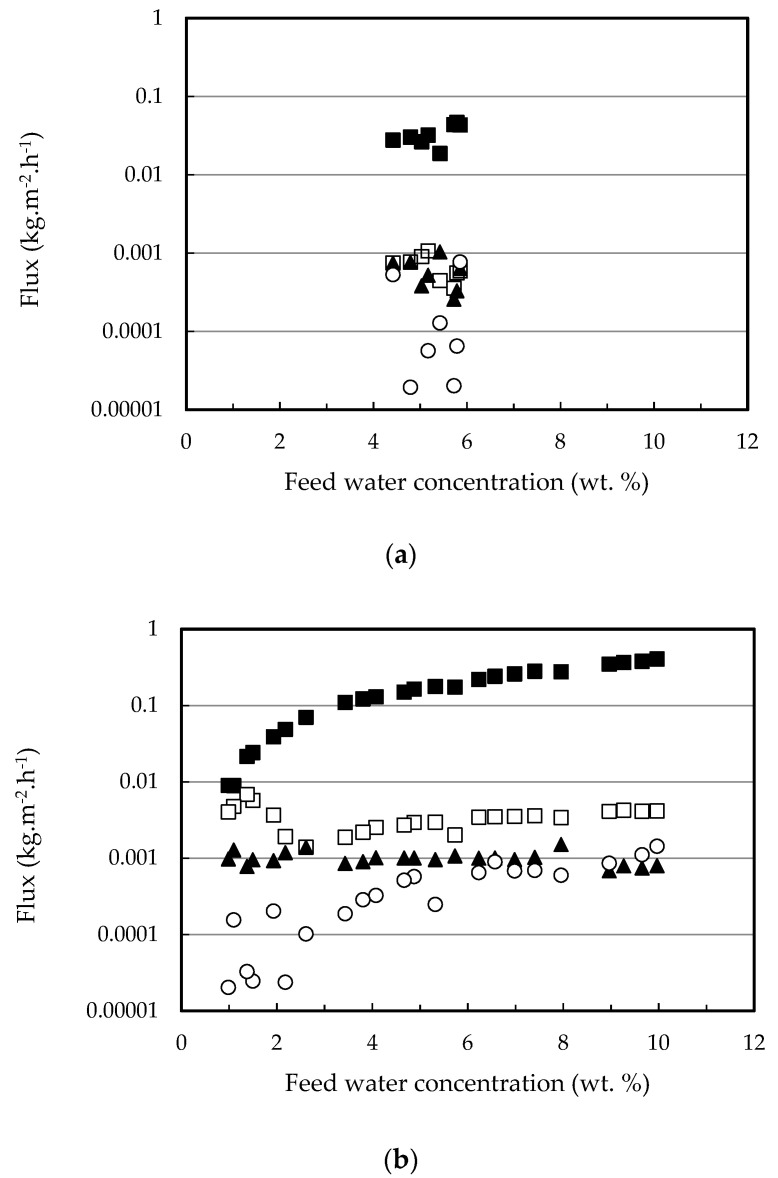

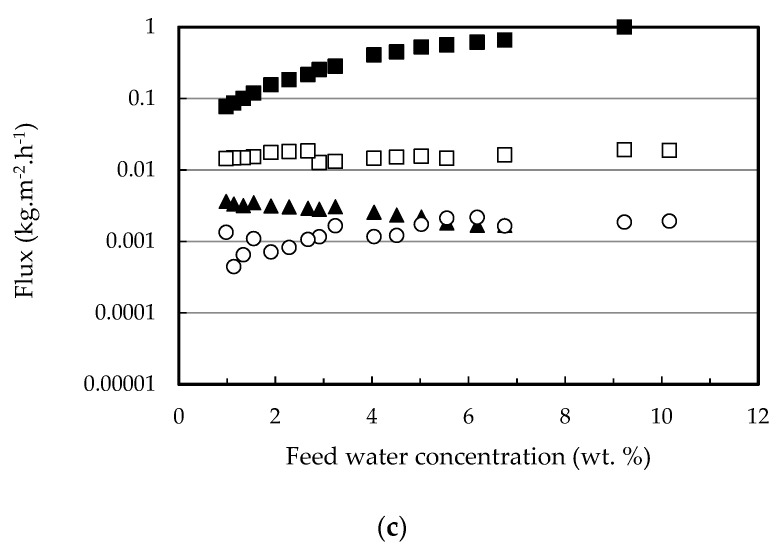
Individual component fluxes as a function of feed water content for the PERVAP™ 1131 membrane at: (**a**) 40 °C, (**b**) 60 °C, (**c**) 80 °C. (■) Water, (☐) ethanol, (▲) ethyl lactate, (○) lactic acid. Initial feed content: 13.5% lactic acid, 17.7% ethyl lactate, 10% water and 58.8% ethanol.

The fluxes of the rest of substances in the quaternary mixture increased with temperature as shown in Figure 9.

**Figure 10:**
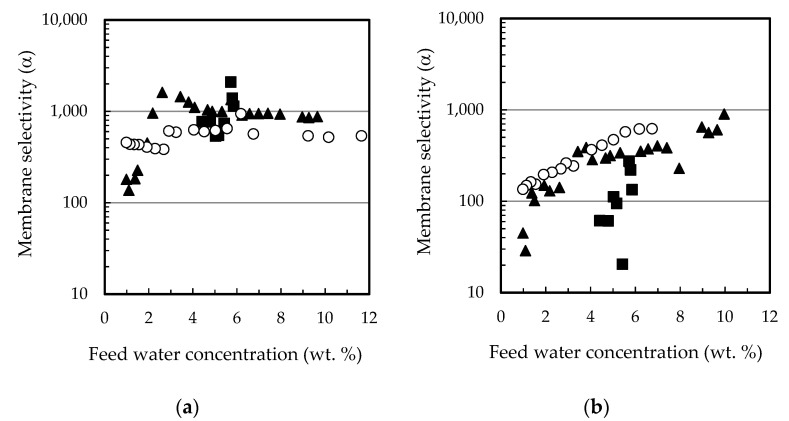
Membrane selectivity as a function of feed water content for the PERVAP™ 1131 membrane at different temperatures: (**a**) water/ethanol, (**b**) water/ethyl lactate. (○) T = 80 °C, (▲) T = 60 °C, (■) T = 40 °C. Initial feed content: 13.5% lactic acid, 17.7% ethyl lactate, 10% water and 58.8% ethanol.

Temperature did not significantly affect water/ethanol selectivity, as shown in Figure 10a, but water/ethyl lactate selectivity showed a higher increase from 40 to 60 °C than from 60 to 80 °C and decreasing as the feed water content was reduced (Figure 10b). Both effects are always in favor of the more volatile component: ethanol in the case of water/ethanol and water in the case of water/ethyl lactate. These effects were expected, since temperature weakens hydrogen bonds and increase the motion of the polymer chains, which facilitates diffusion. The lactic acid is more soluble at higher temperatures, so it crystallizes at lower water concentration, and this allows the membrane to perform better [40].

The dependence of the total permeation flux at any water feed percentage, *J*, with the absolute temperature is described by the formula:

(9) $$J=J_{o}\;exp(-E_{p}/RT)$$

where $J_{o}$ is the pre-exponential factor and $E_{p}$ the activation energy. $J_{o}$ changes with the feed composition, but $E_{p}$ should remain constant. The calculated activation energy between 5.85 and 4.41 wt. % of water was 106.55 kJ/mol, a value in the same order of magnitude but twice the one given by Delgado et al. [41] with the PERVAP™ 2201 from 12 to 63 wt. % of water in the feed.

## 4. Conclusions

Pervaporation membranes PERVAP™ 2216, PERVAP™ 3100 and PERVAP™ 1131 were tested in the dehydration of a mixture of 13.5 wt. % lactic acid, 17.7 wt. % ethyl lactate, 10 wt. % water and 58.8 wt.% ethanol at 60°C. PERVAP™ 1131 was the most appropriate for the process. PERVAP™ 3100 had chemical resistance problems and poor selectivity, and the water fluxes of PERVAP™ 2216 were extremely low.

The effect of feed composition, swelling and temperature effects were studied for the PERVAP™ 1131 membrane. Binary and ternary mixtures have comparable water, ethyl lactate and lactic acid fluxes but lower water/ethanol selectivity than the quaternary one. Lactic acid together with ethyl lactate plays a key role in the water/ethanol selectivity due to the higher affinity of the membrane for them. An increase of temperature increases the permeation fluxes of all substances and increases the passage of the more volatile component in the water/ethanol and water/ethyl lactate systems. Lactic acid is the more soluble component in the membrane, but the one with the lowest vapor pressure, leading to crystallization on the permeate side of the membrane at very low water concentrations, where the nonaqueous environment enhances the crystallization.

## Figures and Tables

**Table 1:** Degree of swelling calculated as the change of weigh of a piece of membrane incubated for 15 days at 60 °C.

Pure Components	Swelling (%)
Ethanol	1.4
Water	7.0
Ethyl lactate	33
Lactic acid	67
**Acidic components**	
Ethanol + 0.5 mL H_2_SO_4_	8.8
Water + 0.5 mL H_2_SO_4_	20
Ethyl lactate + 0.5 mL H_2_SO_4_	60
**Mixtures**	
10% water in ethanol	2.9
17.7% ethyl lactate + 10% water in ethanol	1.4
13.5% lactic acid + 10% water in ethanol	12
17.7% ethyl lactate + 13.5% lactic acid + 10% water in ethanol	15

## Data Availability

Data available upon request to the corresponding author.

## Appendix A

The vapor pressure of pure substances was calculated using an extended Antoine equation, where the temperature *T* is given in K and the vapor pressure $P^{S}$ in Pa:

(A1) $$lnP^{S}=k_{1}+\frac{k_{2}}{T}+k_{3}lnT+k_{4}T^{k_{5}}$$

The parameters for the compounds are given in Table A1. *T_min_* and *T_max_* indicate the minimum and maximum temperature for which the expression is appropriate for each compound (in *K*, as well).

**Table A1:** Extended Antoine equation parameters.

Parameter	Lactic Acid	Ethanol	Water	Ethyl Lactate
*k*_1_	225.19	73.304	73.469	78.774
*k*_2_	−18757	−7122.3	−7258.9	−6715.3
*k*_3_	−28.816	−7.1424	−7.3037	−9.5666
*k*_4_	1.3 × 10^−5^	2.89 × 10^−6^	4.17 × 10^−6^	0.014993
*k*_5_	2	2	2	1
*T_min_*	289.9	159.05	273.16	247.15
*T_max_*	675	514	647.13	588

The UNIQUAC parameters needed for the activity coefficient calculations are given in Table A2 and Table A3.

**Table A2:** UNIQUAC volume and area parameters.

Parameter	Lactic Acid	Ethanol	Water	Ethyl Lactate
***r_i_***	3.1648	2.1055	0.92	4.4555
***q_i_***	2.88	1.972	1.4	3.928

**Table A3:** UNIQUAC binary interaction parameters.

***a_ij_***	**Lactic Acid**	**Ethanol**	**Water**	**Ethyl Lactate**
**Lactic acid**	--	−43.32	155.18	125.29
**Ethanol**	191.28	--	728.97	−148.67
**Water**	−39.61	−756.95	--	64.53
**Ethyl lactate**	52.64	341.77	99.8	--
***b_ij_***	**Lactic acid**	**Ethanol**	**Water**	**Ethyl lactate**
**Lactic acid**	--	0	0	0
**Ethanol**	0	--	−2.0046	0
**Water**	0	2.4936	--	0
**Ethyl lactate**	0	0	0	--
